# Essential oil constituents and variations in antioxidant compounds of dried summer savory (*Satureja* *hortensis* cv. Saturn) affected by storage conditions and ammonium sulfate

**DOI:** 10.1002/fsn3.2451

**Published:** 2021-07-09

**Authors:** Saeideh Mohtashami, Mesbah Babalar, Leila Tabrizi, Askar Ghani, Vahid Rowshan, Majid Shokrpour

**Affiliations:** ^1^ Department of Horticultural Science College of Agriculture Jahrom University Jahrom Iran; ^2^ Department of Horticultural Science Faculty of Agriculture Science and Engineering College of Agricultural and Natural Resources University of Tehran Karaj Iran; ^3^ Department of Natural Resources, Fars Agricultural and Natural Resources Research and Education Center AREEO Shiraz Iran

**Keywords:** ammonium sulfate, carvacrol, essential oil, rosmarinic acid, storage temperature

## Abstract

Considering the importance of spice plants and their shelf life, as affected by various factors, the current study considered Summer savory plants (*Satureja hortensis* cv. Saturn) for evaluation under the application of different concentrations of ammonium sulfate (0, 40, 60, 80, and 100 kg/ha) as primary treatments. Based on the plant response, the control group and 100 kg/ha ammonium sulfate were selected as suitable treatments for storage experiments (i.e., storage at ambient, refrigerator, and freezer temperatures for 8 months). Based on the results, the highest percentage and yield of *S. hortensis* essential oil and biomass occurred in response to 100 kg ammonium sulfate, whereas the lowest amounts were observed in the control group (i.e., in the absence of ammonium sulfate). During the storage period, the essential oil content decreased, but the carvacrol content of the essential oil increased. During the different durations and conditions of storage, the stability of secondary metabolites varied. Essential oil, rosmarinic acid, and carvacrol contents maintained greater stability in plants treated with ammonium sulfate (100 kg/ha), compared with the control group during the storage period. It can be concluded that the preharvest application of ammonium sulfate on *S. hortensis* improved plant growth and quality indices at preharvest time, while also maintaining the stability of its active ingredients at the postharvest stage and storage time. It also led results to recommend storing Summer savory in the freezer to better preserve its secondary metabolites.

## Graphical abstract

During the storage period, essential oil content was reduced but carvacrol content in the essential oil was increased. Ammonium sulfate causes essential oil, Rosmarinic acid, and carvacrol stability during storage. Keeping in a freezer is a good way to summer savory storage.

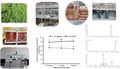

## 1 INTRODUCTION

Summer savory (*Satureja hortensis*, Lamiaceae) is a well‐known medicinal, aromatic, and spice plant that has specific applications in the food and pharmaceutical industries. In addition to high nutritional values, it contains secondary metabolites that are widely used. The vegetative organs of summer savory are effective in increasing blood pressure, cough relief and in being used as a carminative for digestion disorders. Its essential oil has antimicrobial properties and prevents the growth of some bacteria (Azizi et al., 2020; Iranpour Mobarakeh et al., 2014). Essential oils of summer savory species which have higher carvacrol and thymol contents usually show stronger antimicrobial effects. Of course, the presence of relatively proper amounts of carvacrol and other components in the essential oil can optimize the antibacterial effect and quality of the oil (Azizi et al., 2020; Mohtashami et al., 2018). Carvacrol has several biological properties, being anti‐infectious, deworming, anti‐inflammatory, analgesic, antioxidant, antibacterial, and antifungal in addition to being a yeast inhibitor (Fierascu et al., 2018; Hamidpour et al., 2014).

The production of active substances is essentially under the control of genetic processes and environmental factors such as soil conditions, climate, fertilization, irrigation, and other agronomic activities, as well as postharvest operations that affect the shelf life of products (Li et al., 2020; Ncube et al., 2012). The proper application of nutrients during plant growth not only contributes to an increase in yield (Yan et al., 2018) but also improves the quantity and quality of secondary metabolites. Nonetheless, details of this correlation are not entirely clear and, thus, exact studies are required to describe how the physiological and biochemical features affect the synthesis of specific metabolites (Liang et al., 2019).

Plants for medicinal and spice purposes are packed after drying and are stored for a relatively long time or are transported to factories (Suvarnakuta et al., 2011). Essential oils and plant organs that contain essential oils under unsuitable storage conditions can undergo physico‐chemical changes (Jafarzadeh et al., 2020). Essential oils can be badly affected by decomposing processes which usually intensify in the presence of heat, oxygen, humidity, and temperature (Bloem et al., 2011; Mohtashami et al., 2018). Studies on changes in the physical and biochemical properties of medicinal plants under various storage conditions are very valuable because they provide information about traditional medicine and its consumers, while being effective in sustainable harvesting techniques. For instance, some plants are used in the treatment of microbial infections and as anti‐inflammatory agents as they maintain their properties beyond the storage time. This often reduces plant waste and helps avoid repeated harvesting (Pandey & Savita, 2017).

Determining the shelf life and optimal storage conditions of various medicinal plants can assist industries and consumers in optimizing their use of these plants. So far, there has been a small number of species among medicinal plants in the said subject of research. In this regard, previous studies have considered *Thymus officinalis* and *Rosmarinus officinalis* (Usai et al., 2011), *Rosa damascena* (Kazaz et al., 2012; Kumar et al., 2013), *Pleurotus ostreatus* (Kortei et al., 2017), *Li* *ppia citriodora* (Ebadi et al., 2017), *Hy* *poxis hemerocallidea*, *Ocimum basilicum* and *Senna petersiana* (Laher et al., 2013), *Salvia fruticosa* (Dincer et al., 2012), and *Camellia sinensis* (L.) (Xu et al., 2019). In most of the studies on plant storage, the plants were freshly stored and, on the contrary, there have been limited amounts of research on dried herbs and spices. Also, few cases of research have so far addressed the effects of pre‐harvest factors on the storage and phytochemical features of dried plants.

Despite the significant effect of pre‐ and postharvest factors on shelf life, physical and biochemical properties of medicinal, aromatic, and spice plants, there is a lack of scientific information in this area of research. Thus, this experiment was conducted with the aim of investigating the effects of ammonium sulfate, as preharvest treatments, on the phytochemical characteristics of *S. hortensis* during different storage times and conditions.

## 2 MATERIALS AND METHODS

### 2.1 Materials

Plant samples were harvested from a cultivar of *S. hortensis* at the Research Station of the Department of Horticultural Science, University of Tehran, Karaj, Iran (altitude 1,320 m, longitude 51°E, latitude 35°48′N). The details of the cultivation operations are provided below. All chemicals and solvents were purchased from Sigma‐Aldrich Company Ltd. Being of HPLC grade, the chemicals were rosmarinic acid, carvacrol, quercetin, and gallic acid.

### 2.2 Methods

#### 2.2.1 Experimental design

This research was carried out in two sections (pre‐ and postharvest) from 2015 to 2016. In the first section, Summer savory cultivation was carried out in the Alborz province (Karaj, Iran). The experiment was conducted in a randomized complete block design with three replications. For this purpose, the seeds of *S. hortensis* cv. “Saturn” were cultivated in 15 plots at an inter‐ and intra‐row spacing of 30 × 20 cm^2^. The plants were reduced in numbers at the 4–6 leaf stages. Two weeks after planting, different levels of ammonium sulfate were applied. These were the control (0), 40, 60, 80, and 100 kg/ha. At full flowering stage, when the plant had developed its maximum amount of essential oil, the measurements were aimed at plant biomass, percentage, yield, and components of essential oils.

The second section was carried out considering the best treatment among the different levels of applied ammonium sulfate. The selected plant samples were dried and then packed in polyethylene–polyamide packages which had been filled with nitrogen gas and made free of oxygen. They were stored in the dark for 8 months at different temperatures [room temperature (21 ± 2°C), refrigerator temperature (4°C) and freezer temperature (−20°C)]. The phytochemical compounds of the samples were evaluated at the beginning of the experiment (no storage), and after 4–8 months of storage. Then, the measurements were aimed at essential oil content and composition, antioxidant activity, total phenolic compounds, flavones and flavonol contents, total flavonoids content, carvacrol, and rosmarinic acid contents.

### 2.3 Measurement of biochemical traits

#### 2.3.1 Essential oil extraction and determination of its percentage and yield

In each replication, 30 g of crushed dry samples was selected. The extraction was carried out by a Clevenger apparatus and by hydro distillation for 3 hr after boiling and under completely identical conditions. The measurements determined the amounts of essential oil yield and, accordingly, the biomass of each treatment was multiplied by the essential oil percentage of the same treatment and was then divided by 100.

#### 2.3.2 Identification of essential oil compounds by gas chromatography/mass spectrometry (GC/MS)

The GC/MS method was used for analyzing the compounds in essential oils. The Agilent Technologies‐5975C device had a column type of HP‐5‐MS, a length of 30 m and an internal diameter of 0.25 mm, as well as a thickness of 0.25 μm. Other conditions of the operation included an initial temperature of 60°C, a temperature rise ratio of 3°C/min, a final temperature of 280°C, a carrier gas of helium with a flow rate of 1 ml/min, temperature of injection chamber, and temperature of detector 280°C. Retention indices were determined using retention times of n‐alkanes (C_8_‐C_25_) that were injected after the volatile oil under the same chromatographic conditions. The retention indices of all components were determined by the use of n‐alkanes as standard. Identifying the spectra involved using a data bank of mass, retention time, Kovats index, and a study of mass spectra per essential component, as well as a pattern of spectral refraction, compared with standard spectra and the use of reputable sources (Adams, 2001).

#### 2.3.3 Extraction and measurement of polyphenols

The extraction of polyphenols was done according to the method of Misan et al., (2011) with some minor modifications. The isolation and measurement of polyphenol content involved using the HPLC device (Agilent Technologies‐1200 series). The device contained a column C_18_ with a length of 150 mm and an internal diameter of 4.6 mm, along with a particle diameter of 5 μm, a DAD detector at 320 and 280 nm, and an oven temperature of 30°C.

##### Measurement of rosmarinic acid

To determine the rosmarinic acid content in the samples, 20 μl of the extract of each sample was removed and injected into the HPLC device. Standard samples of rosmarinic acid were made at appropriate concentrations (0–500 mg/L) and injected into the HPLC device ultimately, the concentrations of rosmarinic acid were calculated according to the standard curve being drawn. The relevant peaks appeared at about 19.16 min at 280 nm.

##### Measurement of carvacrol

Carvacrol was measured similar to the method used for measuring rosmarinic acid. The concentration of carvacrol in each sample was calculated based on the standard curve (0–500 mg/L). The relevant peak appeared in about 28.49 min at 280 nm.

### 2.4 Extraction and measurement of other biochemical traits

Extraction was performed according to Wojdylo et al., (2007) by maceration and using 70% methanol solvent for the measurement of other biochemical factors.

#### 2.4.1 Determination of antioxidant activity

Antioxidant activity was measured by the “free radical scavenging method” (DPPH) according to Oke et al., (2009). The percentage of antioxidant activity was calculated using the following formula:

$$\text{Antioxidant activity percentage}=\frac{\left(A_{\text{blank}}‐A_{\text{sample}}\right)}{A_{\text{blank}}}\times 100$$

*A*_blank_ is the control absorption number, and *A*_sample_ is sample absorption number.

#### 2.4.2 Measurement of flavones and flavonols, and total flavonoids contents

Flavones and flavonol contents were measured according to a method used by Popova et al., (2004). The results were expressed as quercetin equivalent in mg/g of dry weight. Total flavonoid contents were determined according to a method used by Menichini et al., (2009). The conversion of data was obtained from the absorption into different concentrations of quercetin by drawing a standard curve (0–400 mg/L), and the results were expressed as quercetin equivalent in mg/g of dry weight.

#### 2.4.3 Determination of total phenolic compounds

Total phenolic compounds were measured by the Folin–Ciocalteu reagent (Wojdylo et al., 2007). Data were converted from absorption into different concentrations of gallic acid by drawing a standard curve (gallic acid concentrations of 0–400 mg/L), and the results were expressed as gallic acid equivalent in mg/g of dry weight.

### 2.5 Statistical analysis

The first experiment was conducted in a randomized complete block design with five different levels of ammonium sulfate (control [0], 40, 60, 80, and 100 kg/ha) in three replications. Statistical analysis was performed by the JMP version 8. The second experiment was carried out as factorial based on a randomized complete block design, which consisted of two factors including nutrition (0 as control and 100 kg/ha ammonium sulfate) and different storage temperatures (room temperature, refrigerator temperature, and freezer temperature). Since all traits were measured three times, the repeated measures analysis (RMA) method was used in the Minitab 18 software. The comparison of mean values was done using Tukey's test at the probability level of 5%.

## 3 RESULTS

### 3.1 The effect of ammonium sulfate on plant biomass, essential oil percentage, yield, and composition

Ammonium sulfate had significant effects on biomass, essential oil percentage, and yield (*p* < .01) (data not shown). The highest levels of biomass, essential oil percentage, and yield were obtained by the application of 100 kg ammonium sulfate (49.17 ± 2.92 g, 3.55 ± 0.33%, and 49.97 ± 4.62 ml, respectively), whereas the lowest contents (19.42 ± 3.89 g, 2.68 ± 0.35%, and 16.32 ± 2.14 ml, respectively) were observed in the control (Table 1). The chemical constituents in Summer savory were identified in its essential oil and were affected by different levels of ammonium sulfate (Table 1). As a major ingredient in *S. hortensis* essential oil, carvacrol reflected many of the properties of the essential oil and was affected by different levels of ammonium sulfate. The application of 40 kg ammonium sulfate resulted in the highest carvacrol content (52.06 ± 2.32%), whereas the lowest content (46.57 ± 4.48%) occurred in response to the 60 kg ammonium sulfate treatment (Table 1). Meanwhile, the maximum content of α‐terpinene (38.22 ± 3.64%) was observed in the control. According to the results, the treatment with 100 kg ammonium sulfate and the control group were finally selected for further applications in the storage experiments.

**TABLE 1:** Effect of different levels of ammonium sulfate on biomass, essential oil percentage, and yield of *S. hortensis* cv. Saturn

Treatments	Biomass (g)	Essential oil percentage (%)	Essential oil yield (ml)	Carvacrol	γ‐Terpinene	α‐Terpinene
**Control (0 Kg)**	19.42 ± 3.89d	2.68 ± 0.35c	16.32 ± 2.14c	47.53 ± 5.61d	38.22 ± 3.64a	3.71 ± 0.50bc
**40 Kg**	25.70 ± 1.08c	2.90±0.09bc	22.84 ± 0.68c	52.06 ± 2.32a	34.85 ± 2.18bc	3.33 ± 0.14c
**60 Kg**	26.97 ± 0.96c	3.03 ± 0.60abc	23.07 ± 4.54c	46.57 ± 4.48e	36.61 ± 3.49a	3.95 ± 0.34ab
**80 Kg**	31.18 ± 2.62b	3.38 ± 0.48ab	31.51 ± 4.43b	48.80 ± 3.37c	36.94 ± 2.63ab	3.76 ± 0.38b
**100 Kg**	49.17 ± 2.92a	3.55 ± 0.33a	49.97 ± 4.62a	49.89 ± 1.91b	34.28 ± 0.95c	4.18 ± 0.13a

Note

Means with similar letter are not significant in%5 level of Tukey test.

### 3.2 Changes in *S. hortensis* secondary metabolites treated with ammonium sulfate under different storage conditions

#### 3.2.1 Essential oil content

**FIGURE 1:**
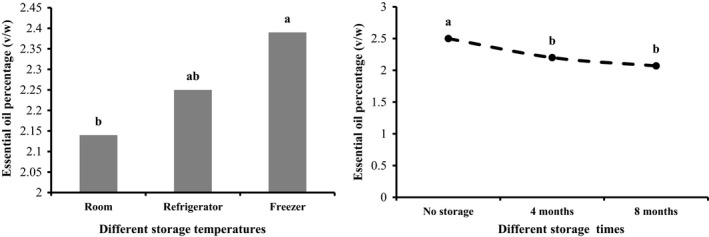
Simple effect of storage temperature and time on *S. hortensis* cv. Saturn essential oil

**FIGURE 2:**
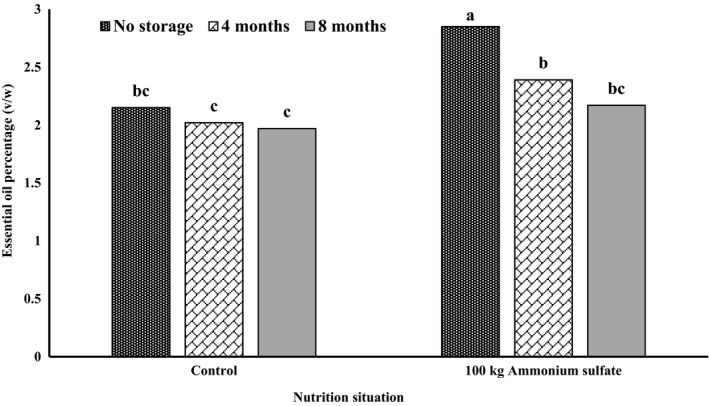
Interaction between ammonium sulfate and storage time on essential oil percentage of *S. hortensis* cv. Saturn

The simple effects of ammonium sulfate, storage temperature, storage time, and interaction between ammonium sulfate and storage time on essential oil content were significant (*p* < .01), while other interactions had no significant effect (data not shown). Regarding storage temperature, the lowest essential oil content (2.14 ± 0.41%) and the highest amount (2.39 ± 0.34%) were observed at room temperature and in the freezer, respectively. In other words, increasing the temperature caused a decrease in the amount of essential oil (Figure 1). Generally, the amount of essential oil decreased over time, so that the highest (2.50 ± 0.42%) and lowest contents (2.07 ± 0.27%) were found in the outset (no storage) and after storage for 8 months, respectively (Figure 1). The interaction between ammonium sulfate and storage time led to the highest essential oil content (2.85 ± 0.18%) as a result of the 100 kg ammonium sulfate in the beginning of storage. This was followed by 100 kg of ammonium sulfate after 4 months of storage. The lowest essential oil content (1.97 ± 0.22%) was extracted from the control group after 8 months of storage. During storage, the amount of essential oil decreased in samples of the 100 kg ammonium sulfate treatment and the control. The samples treated with ammonium sulfate during the 4‐month storage showed greater stability (Figure 2).

#### 3.2.2 Essential oil components

The chemical constituents in Summer savory essential oil were affected by the treatments. Several of the most important compounds were α‐Thujene, α‐Pinene, Myrcene, α‐Terpinene, ρ‐Cymene, β‐Phellandrene, γ‐Terpinene, and carvacrol.

##### α‐Thujene

The highest α‐thujene (1.56 ± 0.14%) was obtained in samples treated with 100 kg of ammonium sulfate at the beginning of storage, which were statistically in the same group as the control. There was no significant difference between the other treatments, so that not applying ammonium sulfate in the 4 months of storage resulted in the lowest amount of α‐Thujene (1.09 ± 0.14%) (Table 2).

**TABLE 2:** The effect of ammonium sulfate and storage time on main compounds of *S. hortensis* cv. Saturn essential oil

	α‐Thujene (%)			α‐Terpinene (%)		
	No storage	4 months	8 months	No storage	4 months	8 months
**Ammonium sulfate**						
**Control**	1.40 ± 0.18a	1.09 ± 0.14b	1.15 ± 0.10b	3.71 ± 0.11b	3.37 ± 0.45c	3.71 ± 0.11b
**100 kg**	1.56 ± 0.14a	1.15 ± 0.01b	1.11 ± 0.05b	4.19 ± 0.44a	3.73 ± 0.22b	3.65 ± 0.11bc

	ρ‐Cymene (%)			γ‐Terpinene (%)		
**Ammonium sulfate**						
**Control**	1.21 ± 0.12c	3.47 ± 0.89a	3.64 ± 0.93a	38.22 ± 0.82a	35.75 ± 2.07b	34.49 ± 1.03b
**100 kg**	2.28 ± 0.94b	2.72 ± 1.86b	3.48 ± 0.80a	34.28 ± 3.15b	35.40 ± 1.19b	33.97±0.81b

Note

Means with similar letter are not significant in%5 level of Tukey test.

##### α‐Pinene

α‐pinene content was affected by the simple effect of storage temperature (*p* < .05) and storage time (*p* < .01) as well as the interaction between ammonium substrate and storage temperature (*p* < .05), while the other interactions were not significant. The maximum content of α‐pinene (0.90 ± 0.18%) was in the samples treated with 100 kg of ammonium sulfate at room temperature. In contrast, the lowest content (0.73 ± 0.15%) occurred in samples treated with ammonium sulfate (100 kg) and which were stored in the refrigerator, so that this treatment was in the same group as the other treatments (Table 3).

**TABLE 3:** The effect of ammonium sulfate and storage temperature on main compounds of *S. hortensis* cv. Saturn essential oil

	α‐Pinene (%)			α‐Terpinene (%)		
	Room	Refrigerator	Freezer	Room	Refrigerator	Freezer
**Ammonium sulfate**						
**Control**	0.79 ± 0.12ab	0.80 ± 0.14ab	0.81 ± 0.11ab	3.59 ± 0.19b	3.72 ± 0.11abc	3.47 ± 0.02c
**100 kg**	0.90 ± 0.18a	0.73 ± 0.15b	0.84 ± 0.17ab	3.84 ± 0.40ab	3.79 ± 0.40ab	3.93 ± 0.33a

Note

Means with similar letter are not significant in%5 level of Tukey test.

##### Myrcene

**FIGURE 3:**
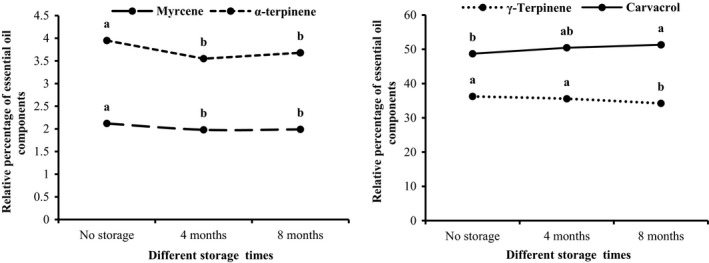
Changes in some main constituents of *S. hortensis* cv. Saturn essential oil during storage time

Myrcene was only affected by the simple effect of storage time (*p* < .05). The highest myrcene content (2.12 ± 0.11%) was measured in the beginning of storage and then showed a decreasing trend through time in storage, so that the lowest content (1.98 ± 0.12%) was observed at the end of storage (8 months). There was no significant difference between the content measured after 4 and 8 months of storage time (Figure 3).

##### α‐Terpinene

The triple effects of ammonium sulfate, storage temperature, and storage time (*p* < .05) were significant on the amount of α‐terpinene (Table 4). The results revealed that the highest amount of α‐terpinene (4.19 ± 0.50%) was detected in samples treated with 100 kg ammonium sulfate in the beginning of storage and at all three storage temperatures. The lowest content (2.97 ± 0.59%), however, was in the control sample after 4 months of storage in the freezer, while the other treatments were in the same statistical group (Table 4).

**TABLE 4:** Interaction of ammonium sulfate and storage time and storage temperature on main compounds of *S. hortensis* cv. Saturn essential oil during 8 months storage

Ammonium sulfate	Storage temperature	α‐Terpinene (%)			β‐Phellandrene (%)		
		No storage	4 months	8 months	No storage	4 months	8 months
**Control**	Room	3.71 ± 0.13ab	3.38 ± 0.03bc	3.69 ± 0.14ab	0.52 ± 0.02a	0.29 ± 0.01 cd	0.28 ± 0.02 cd
	Refrigerator	3.71 ± 0.13ab	3.76 ± 0.04ab	3.70 ± 0.16ab	0.52 ± 0.02a	0.26 ± 0.07d	0.30 ± 0.02 cd
	Freezer	3.71 ± 0.13ab	2.97 ± 0.59c	3.74 ± 0.02ab	0.52 ± 0.02a	0.27 ± 0.03 cd	0.34 ± 0.03c
**100 kg**	Room	4.19 ± 0.50a	3.66 ± 0.36ab	3.68 ± 0.03ab	0.49 ± 0.07ab	0.22 ± 0.02d	0.39 ± 0.09c
	Refrigerator	4.19 ± 0.50a	3.62 ± 0.01abc	3.57 ± 0.16abc	0.49 ± 0.07ab	0.25 ± 0.04d	0.27 ± 0.01 cd
	Freezer	4.19 ± 0.50a	3.90 ± 0.01ab	3.70 ± 0.10ab	0.49 ± 0.07ab	0.26 ± 0.01d	0.28 ± 0.03 cd

Note

Means with similar letter are not significant in%5 level of Tukey test.

##### ρ‐Cymene

**FIGURE 4:**
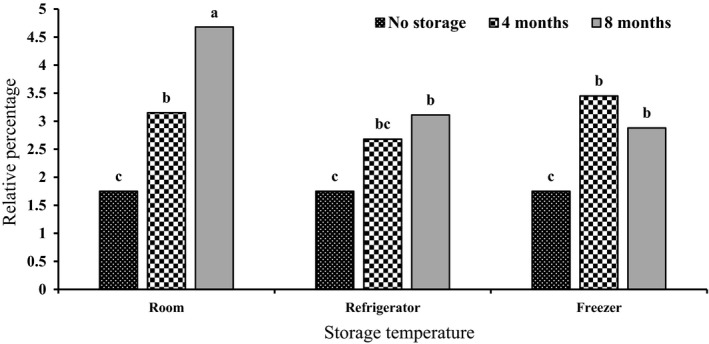
Changes in ρ‐Cymene content of *S. hortensis* cv. Saturn essential oil effected by storage time and storage temperature

The simple effects of storage temperature (*p* < .01), storage time (*p* < .01), interaction between ammonium sulfate, and storage time (*p* < .01), as well as the interaction between storage temperature and storage time (*p* < .01), were significant on ρ‐cymene. The highest levels of ρ‐cymene (3.64 ± 0.93%) were observed in the control after 8 months of storage. The lowest amount (1.21 ± 0.12%) was observed in the control at the beginning of storage (Table 2). After 8 months of storage at room temperature, the highest amount of ρ‐cymene (4.68 ± 0.25%) was obtained, whereas the lowest content (1.75 ± 0.90%) occurred in the beginning of storage and at all three storage temperatures (Figure 4).

##### β‐Phellandrene

According to the results, the highest β‐Phellandrene content (0.52 ± 0.02%) was obtained in the beginning of storage and in the control treatment among all three storage conditions. In contrast, the lowest content (0.22 ± 0.02%) was measured in samples treated with 100 kg of ammonium sulfate after 4 months of storage at room temperature (Table 4).

##### γ‐Terpinene

All the simple effects (ammonium sulfate, storage temperature, and storage time) (*p* < .01) and the interaction between ammonium sulfate and storage time (*p* < .01) significantly affected the amount of γ‐terpinene. The highest amount of γ‐terpinene content (38.22 ± 0.82%) was found in the control at the beginning of storage and, thereafter, the other treatment groups were categorized in the same statistical group. The lowest content (33.97 ± 0.81%) was observed in the treatment group of 100 kg ammonium sulfate after 8 months of storage. As a result, γ‐Terpinene decreased in the control samples over time, whereas in samples treated with 100 kg ammonium sulfate, there was an initial, slight decrease (after 4 months of storage), followed by a further decrease through the 8 months of storage (Table 2).

##### Carvacrol

According to the results of analysis of variance, only the simple effect of storage time (*p* < .01) was significant on the carvacrol content, whereas the other simple effects and interactions were not significant. The amount of carvacrol increased over time so that the highest (51.31 ± 1.00%) was measured at the end of storage (8 months) and the lowest content (48.71 ± 3.72%) was observed at the beginning of storage (Figure 3).

#### 3.2.3 Flavones and flavonols contents

By using 100 kg ammonium sulfate and by storage at freezer temperatures in the beginning of the storage period, the results led to the highest amounts of flavones and flavonols (20.11 ± 1.32 mg/g). However, this treatment was in the same group with other treatments at the beginning of storage. The next in value was the control treatment after 8 months of storage in the refrigerator (17.28 ± 1.05 mg/g). The lowest amounts (2.8 ± 0.63 mg/g) were observed as a result of the 100 kg ammonium sulfate treatment on samples stored in the freezer after 8 months (Table 5).

**TABLE 5:** Interaction of ammonium sulfate and storage time and storage temperature on some phytochemical compounds of *S. hortensis* cv. Saturn extract during 8 months storage

Ammonium sulfate	Storage temperature	Flavones and flavonols (mg/g)			Flavonoids content (mg/g)		
		No storage	4 months	8 months	No storage	4 months	8 months
**Control**	Room	18.30 ± 1.29a	11.9 ± 1.43de	9.54 ± 0.85def	19.32 ± 2.55de	10.13 ± 1.59h	18.18 ± 1.27d‐g
	Refrigerator	18.19 ± 1.27a	11.39 ± 1.63de	17.28 ± 1.05ab	19.6 ± 2.29de	14.73 ± 0.84fg	48.37 ± 1.43a
	Freezer	18.10 ± 1.96a	13.43 ± 1.61abc	5.71 ± 0.83fg	19.28 ± 1.93de	14.53 ± 0.56g	18.65 ± 1.05def
**100 kg**	Room	20.04 ± 0.65a	11.91 ± 2.54cde	9.75 ± 0.39def	33.6 ± 1.51b	16.33 ± 1.37d‐g	17.02 ± 1.49d‐g
	Refrigerator	19.95 ± 2.02a	12.17 ± 0.35cde	9.14 ± 1.05ef	34.1 ± 2.07b	20.05 ± 1.83d	33.25 ± 0.95b
	Freezer	20.11 ± 1.32a	16.1 ± 0.76abc	2.8 ± 0.63g	33.26 ± 1.11b	24.28 ± 1.42c	15.94 ± 0.49efg

Ammonium sulfate	Storage temperature	Total phenolic compounds (mg/g)			Antioxidant activity (%)		
		No storage	4 months	8 months	No storage	4 months	8 months
**Control**	Room	12.77 ± 1.92f–h	12.51 ± 1.39gh	14.15 ± 1.95e–h	74.08 ± 3.09c	39.45 ± 0.98c	23.63 ± 5.12de
	Refrigerator	12.87 ± 0.33f–h	16.34 ± 1.92d–g	24.44 ± 1.28a	77.90 ± 3.56a	43.77 ± 2.44bc	27.56 ± 5.34d
	Freezer	12.84 ± 0.33f–h	10.32 ± 0.53h	13.67 ± 1.64e–h	75.67 ± 6.21a	51.77 ± 3.11b	6.60 ± 0.90f
**100 kg**	Room	20.76 ± 0.74ab	13.27 ± 2.36f–h	16.48 ± 1.50c–g	71.90 ± 4.43a	39.06 ± 2.01c	16.30 ± 0.78ef
	Refrigerator	20.67 ± 1.40abc	16.81 ± 1.77b–f	19.97 ± 1.65bcd	73.48 ± 4.56a	38.75 ± 1.80c	13.48 ± 1.70ef
	Freezer	20.72 ± 1.05ab	17.67 ± 1.46b–e	10.75 ± 1.21h	72.48 ± 2.55a	50.83 ± 2.54b	11.07 ± 1.25f

Note

Means with similar letter are not significant in%5 level of Tukey test.

#### 3.2.4 Total flavonoids content

The highest amount of flavonoids (48.37 ± 1.43 mg/g) was observed in the control group and in the refrigerator after 8 months of storage. The lowest content (10.13 ± 1.59 mg/g) was found in the control at room temperature after 6 months of storage (Table 5). The amounts of flavonoids in samples of both the control and the 100 kg ammonium sulfate treatment group showed a decreasing trend and then an increasing one, so that the increase in the control sample was higher than that of the 100 kg ammonium sulfate treatment group.

#### 3.2.5 Total phenolic compounds

The highest amount of phenolic compounds (24.44 ± 1.28 mg/g) was observed in the control group after 8 months of storage in the refrigerator. The lowest content (10.32 ± 0.53 mg/g) was measured in the control after 4 months of storage in the freezer, which was not significantly different from the 100 kg ammonium sulfate treatment group stored in the freezer after 8 months (Table 5). The amount of phenolic compounds in the control samples increased over time, whereas the samples treated with 100 kg of ammonium sulfate showed a decreasing trend.

#### 3.2.6 Antioxidant activity percentage

The highest antioxidant activity (77.90 ± 3.56%) was found in the control group at the beginning of storage, which was not significantly different from other treatment groups at the same stage. The lowest antioxidant activity (6.60 ± 0.90%) was observed in the control being in the freezer at the end of storage time, which was in the same group with 100 kg of ammonium sulfate treatment stored in the freezer at the end of storage time (Table 5). These results indicated that the refrigerator temperature was better for storage of control samples, whereas it was not an appropriate option for storage of samples treated with 100 kg of ammonium sulfate. The latter were best stored in the freezer. The antioxidant activity in both the control and 100 kg ammonium sulfate treatment groups ultimately reached zero. This reduction in control samples was higher than samples treated with ammonium sulfate (Table 5). The antioxidant activity at all three room temperatures, refrigerator, and freezer decreased over time. This decrease was greater at the freezer temperature, but was least at room temperature.

#### 3.2.7 Rosmarinic acid and carvacrol content in plant extracts

The interaction effect between ammonium sulfate and storage temperature resulted in the highest rosmarinic acid content (6.51 ± 1.39 mg/g dry weight) in samples of 100 kg ammonium sulfate treatment at room temperature. The lowest content (4.54 ± 1.44 mg/g) was observed in the control at room temperature. This suggests that treating the samples with ammonium sulfate could have a positive effect on the stability of rosmarinic acid and showed different results compared with untreated plants. Room temperature storage was more appropriate for samples treated with 100 kg of ammonium sulfate than the refrigerator and freezer temperatures. In control samples, however, the result was reversed and storage at room temperature was not appropriate (Table 6). The interaction between ammonium sulfate and storage time showed that the highest amount of rosmarinic acid (6.91 ± 1.00 mg/g) was obtained in response to 100 kg ammonium sulfate after 4 months of storage. The lowest content (4.31 ± 1.16 mg/g) was observed in the control after 8 months of storage. The amount of rosmarinic acid in the control samples decreased over time. Compared with the control and with ammonium sulfate samples of 8‐month storage, samples treated with 100 kg ammonium sulfate retained more rosmarinic acid.

**TABLE 6:** The effect of ammonium sulfate and storage temperature and storage time on rosmarinic acid and carvacrol constituents of *S. hortensis* cv. Saturn extract

	Rosmarinic acid (mg/g)			Carvacrol (mg/g)		
	Room	Refrigerator	Freezer	Room	Refrigerator	Freezer
**Ammonium sulfate**						
Control	4.54 ± 1.44c	5.73 ± 0.72abc	5.21 ± 1.20bc	3.26 ± 0.72c	4.26 ± 0.58ab	3.86 ± 0.62bc
100 kg	6.51 ± 1.39a	6.07 ± 0.90ab	5.91 ± 0.98b	4.88 ± 0.79a	4.74 ± 0.70a	4.27 ± 0.42ab

	No storage	4 months	8 months	No storage	4 months	8 months
**Ammonium sulfate**						
Control	6.13 ± 0.79abc	5.04 ± 0.98 cd	4.31 ± 1.16d	4.02 ± 0.55bc	3.76 ± 0.75bc	3.60 ± 0.91c
100 kg	6.27 ± 1.05ab	6.91 ± 1.00a	5.31 ± 0.61bcd	4.33 ± 0.49b	5.15 ± 0.80a	4.42 ± 0.37b

Note

Means with similar letter are not significant in %5 level of Tukey test.

**FIGURE 5:**
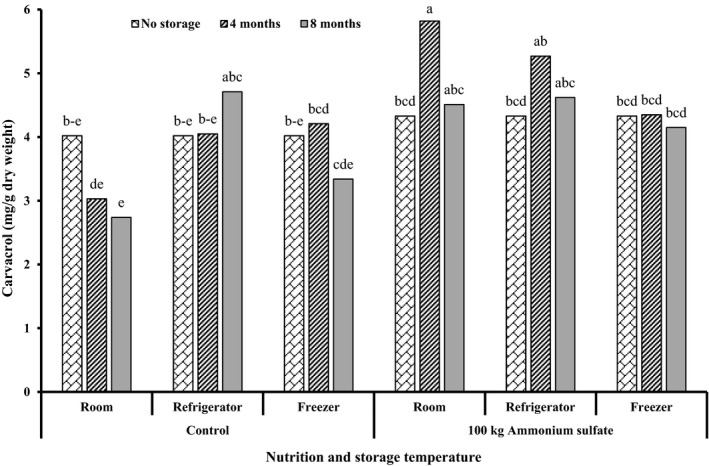
Triple effects of nutrition, storage temperature, and storage time of carvacrol content of summer savory (*S. hortensis* cv. Saturn) extract

The results of analysis of variance showed the significant effects of all simple and interaction effects on carvacrol content, except in the case of interaction between storage temperature and storage time. The highest amount of carvacrol (5.82 ± 0.43 mg/g dry weight) occurred in response to 100 kg ammonium sulfate at room temperature after 4 months of storage. The lowest carvacrol content (2.74 ± 0.31 mg/g) was obtained in the control at room temperature after 8 months of storage (Figure 5). The interaction effects between ammonium sulfate and storage temperature led to the highest amount of carvacrol (4.88 ± 0.79 mg/g) in response to the 100 kg ammonium sulfate at room temperature, which was not significantly different from the 100 kg ammonium sulfate in the refrigerator. The lowest content (3.26 ± 0.72 mg/g) was observed in the control at room temperature. The response of carvacrol and rosmarinic acid to the interaction between ammonium sulfate and storage temperature was similar (Table 6). The results of the interaction between ammonium sulfate and storage time showed that the application of 100 kg ammonium sulfate within a timeframe of 4 months of storage resulted in the highest amount of carvacrol (5.15 ± 0.80 mg/g), whereas the lowest content (3.60 ± 0.91 mg/g) was found in the control group after 8 months of storage. These results were similar to those of rosmarinic acid. Carvacrol levels in the control samples decreased over time, whereas it increased in samples treated with ammonium sulfate, so that the increase was more notable through the 4 months of storage than through other storage periods.

## 4 DISCUSSION

There are various reports indicating that the quality of medicinal plants varies with different environmental factors (pre‐ and postharvest). For example, essential oil contents decreased over time in medicinal plants during storage. This condition was reported in the case of *Lippia citriodora* (Ebadi et al., 2017), *Rosa damascena* (Kumar et al., 2013), and *Ocimum basilicum* (Rosado et al., 2013), but the rate of decrease was slight in cold storage. Studies on biological activity of 19 medicinal plants from Nepal after 6 years of storage showed that three plants lost their antibacterial and antifungal activity, while six plants fully maintained their biological activity and 10 plants maintained part of their activity (Griggs et al., 2001). It has also been shown that during storage of medicinal plants, their active ingredients, including essential oils, are affected by the type and method of packaging, as well as by storage temperature, so that shortening the storage time, reducing the temperature, and using barrier packaging was effective in maintaining the quality of the essential oil (Ebadi et al., 2017; Jesus et al., 2016; Lisboa et al., 2018). The results of the present study revealed that the difference in active substances showed different responses to ammonium sulfate, storage temperature, and storage time. Therefore, it appeared more appropriate to feed plants with 100 kg ammonium sulfate and to store them in the freezer in order to preserve the essential oil of *S. hortensis* efficiently. In addition, the amount of essential oil during storage decreased over time. Due to the fact that Summer savory essential oil is made and stored in the hairy cuticle and secretory glands on the leaf surface, it is exogenous and highly volatile. Thus, it is more susceptible to destruction and reduction than other active ingredients. Due to the breakage and damage of the essential oil glands during the process of drying and packaging, associated with further evaporation, the glands are reduced in numbers during storage. Therefore, storage at lower temperatures, such as in the freezer, can lead to a reduction in the evaporation of essential oils and cause greater stability (Mohtashami et al., 2018; Rehman et al., 2016). Ebadi et al., (2017) reported oxidative reactions and evaporation as a reason for the decrease in essential oil during storage. So, it seems that storage for 4 months can result in better outcomes, compared with storage for 8 months, due to its proper effects on essential oil content and maintenance.

Regarding the essential oil components, different compounds exhibited different responses, so that some compounds increased while others decreased during storage. The most important components of Summer savory essential oil were carvacrol and γ‐Terpinene, which were present in higher amounts in the essential oil than other compounds and determined the quality index of Summer savory essential oil. Carvacrol was not affected by ammonium sulfate and storage temperature. In fact, it only increased over time, whereas γ‐Terpinene decreased. Essential oils are composed of various components that are measured as relative percentages. Some of these compounds are precursors to other compounds. This suggests that the precursor of carvacrol (i.e., γ‐terpinene) converts to carvacrol based on enzymatic or non‐enzymatic interactions that take place during the storage of essential oil (Mohtashami et al., 2018). Researchers have described changes in the amount of essential oil components, water activity, and available water content in products, as well as the availability of oxygen and different temperatures during the storage period (Ebadi et al., 2017). The most important factors in maintaining the quality of medicinal plants are water and oxygen transmission rates. The penetration of some volatile compounds into the packaging material is another reason for changing or reducing some components of the essential oil (Chaliha et al., 2013). Changes in essential oil components during storage depend on the type of composition, plant species, and storage conditions (Mahmoodi sourestani et al., 2014). The biotransformation of terpenes can also indicate the occurrence of these changes, so that terpenes are able to bind or release water molecules in order to isomerize or rearrange, while essential oil components themselves or trace contaminants may catalyze or initiate these reactions (Misharina, 2001).

The amounts of flavones and flavonols decreased over time, and this decrease was greater at the freezer temperature. Meanwhile, they were more stable at the refrigerator temperature. In addition, more flavones and flavonols were retained in the control samples, compared with those treated with 100 kg of ammonium sulfate. After 8 months of storage, the control samples in the refrigerator were the most stable. However, after 4 months of storage, the samples treated with 100 kg ammonium sulfate in the freezer were the most stable. The flavonoid content decreased after 4 months of storage, compared to the beginning of storage, and then increased until 8 months of storage. The increase in flavonoid contents of the control samples in the refrigerator was higher than in other treatment groups. Flavonoids were more stable during shorter storage periods (4 months) in samples treated with 100 kg ammonium sulfate in the freezer. Phenolic compounds were more stable in the control samples stored in the refrigerator, while their rate increased with time. However, the samples treated with 100 kg ammonium sulfate had a decrease in phenolic compounds over time. Freezer storage negatively affected the amount of phenolic compounds. The antioxidant activity was also higher in control samples than in samples treated with 100 kg ammonium sulfate, although it decreased over time and in contrast to phenolic compounds. Storage in the freezer was the best treatment for 4 months of storage, while 8 months of storage had an adverse effect and caused the lowest antioxidant activity, especially in the freezer storage temperature. In longer storage periods (8 months), storing in the refrigerator preserved antioxidant activity better than in other conditions. Rosmarinic acid and carvacrol were more stable in samples treated with 100 kg ammonium sulfate at room temperature. Rosmarinic acid content decreased over time but the carvacrol content increased.

The results of this study showed that some compounds, such as essential oil, flavones and flavonols, antioxidant activity, and rosmarinic acid, decreased over time. This is consistent with the results of previous research (Javadi et al., 2015; Kazaz et al., 2012; Meng Li et al., 2015; Ryu et al., 2011). According to the results of the present research and other similar cases of research, the reason for the reduction in these active substances can be due to the inaccessibility of nutrients at postharvest time or because of compound degradation, since nutrients are responsible for the biosynthesis of active substances by microbial or enzymatic activity (Jafarzadeh et al., 2020; Ryu et al., 2011). It should be noted that, in this study, some compounds such as flavonoids, phenolic compounds, and carvacrol increased in the essential oil and extract over time. This effect was also reported in plants such as soybeans (Prabakaran et al., 2018) and Cornelian Cherry (Moldovan et al., 2016) for which researchers proposed reasons such as a continued release of these compounds from the plant matrix, or the degradation of complex polymerized phenolic structures to simple structures during storage.

## 5 CONCLUSION

The importance of essential oil, carvacrol, and rosmarinic acid makes them qualitative indicators of this plant. According to the results of this study, it can be concluded that the pre‐harvest application of ammonium sulfate in *S. hortensis* not only improves plant growth and quality indices at pre‐harvest time, but also improves the stability of its active substances at the postharvest stage and during storage. With ammonium sulfate and with freezer temperatures, the storage can lead to a better preservation of important substances in the essential oil.

## CONFLICT OF INTEREST

The authors declare that they have no known competing financial interests or personal relationships that could have appeared to influence the work reported in this paper.

## Data Availability

The data that support the findings of this study are available from the corresponding author upon reasonable request.
